# R-loops and initiation of DNA replication in human cells: a missing link?

**DOI:** 10.3389/fgene.2015.00158

**Published:** 2015-04-28

**Authors:** Rodrigo Lombraña, Ricardo Almeida, Alba Álvarez, María Gómez

**Affiliations:** Functional Organization of the Genome Group, Centro de Biología Molecular “Severo Ochoa”, Consejo Superior de Investigaciones Científicas/Universidad Autónoma de Madrid, Madrid, Spain

**Keywords:** R-loops, CpG islands, ORC, G-quadruplex, DNA replication origins

## Abstract

The unanticipated widespread occurrence of stable hybrid DNA/RNA structures (R-loops) in human cells and the increasing evidence of their involvement in several human malignancies have invigorated the research on R-loop biology in recent years. Here we propose that physiological R-loop formation at CpG island promoters can contribute to DNA replication origin specification at these regions, the most efficient replication initiation sites in mammalian cells. Quite likely, this occurs by the strand-displacement reaction activating the formation of G-quadruplex structures that target the origin recognition complex (ORC) in the single-stranded conformation. In agreement with this, we found that R-loops co-localize with the ORC within the same CpG island region in a significant fraction of these efficient replication origins, precisely at the position displaying the highest density of G4 motifs. This scenario builds on the connection between transcription and replication in human cells and suggests that R-loop dysregulation at CpG island promoter-origins might contribute to the phenotype of DNA replication abnormalities and loss of genome integrity detected in cancer cells.

R-loops are three-stranded nucleic acid structures formed upon the hybridization of an RNA strand to a complementary DNA strand. This RNA/DNA hybrid displaces the second DNA strand into a looped out state, giving this class of structures their name. *In vivo*, R-loops can be generated by RNA polymerase II transcribing a C-rich DNA template such that a G-rich transcript is produced. Although the mechanism through which R-loops are generated is still unclear, the prevalent model postulates that the newly synthesized RNA strand, upon leaving the RNA exit channel of the traveling RNA polymerase complex, competes with the non-template DNA strand for re-annealing to the template DNA strand. Once formed, R-loops are stable, as RNA/DNA interactions are thermodynamically far more stable than the corresponding DNA/DNA duplexes (Roberts and Crothers, 1992).

R-loops were first detected *in vivo* at prokaryotic ORIs (Masukata et al., 1987; Baker and Kornberg, 1988; Masukata and Tomizawa, 1990; Carles-Kinch and Kreuzer, 1997), the mitochondrial origin of replication (Xu and Clayton, 1996), the immunoglobulin class-switch region in activated B cells (Yu et al., 2003), and in yeast cells mutant for mRNA metabolism genes (Huertas and Aguilera, 2003). More recently, genome-wide approaches to measure R-loops showed that these nucleic acid structures are widespread in the human genome, being prevalently formed at promoter 5′- and terminator 3′-end regions of several genes (Ginno et al., 2012, 2013). R-loops are involved in multiple cellular processes including transcription repression, transcriptional termination, DNA methylation and histone modifications, as well as DNA replication and immunoglobulin class switch recombination. Importantly, unprogrammed R-loop formation or R-loop dysregulation can promote DNA damage and genome instability that may lead to human diseases, placing this nucleic acid structure at the center of very active research in recent years (see the excellent reviews, by Aguilera and García-Muse, 2012; Groh and Gromak, 2014; Skourti-Stathaki and Proudfoot, 2014). Curiously, the aspect of R-loop biology that has been overlooked in recent investigations is their role in DNA replication initiation, which was actually the first biological function ascribed for R-loops more than 30 years ago. In this article, we revisit this issue and propose that, in human cells, persistent R-loop formation can play a role in replication origin specification likely through exposing and activating replication signals that are functional only in the single-stranded conformation by the strand-displacement reaction.

## R-loops and Initiation of DNA Replication

The earliest evidence for a role for R-loops in initiation of DNA replication came from studies in the late 1980’s on *Escherichia coli* plasmid ColEI. In this system, a transcript initiated from an upstream promoter forms a persistent hybrid with the template strand within specific origin elements (Dasgupta et al., 1987; Masukata and Tomizawa, 1990). The hybridized RNA is cleaved by RNAse H and then serves as a primer for DNA synthesis by DNA polymerase I (Itoh and Tomizawa, 1980). In the absence of RNAase H, the persistent DNA-RNA hybrid indirectly activates subsequent DNA synthesis instead of providing a primer as it occurs in the presence of the enzyme (Masukata et al., 1987). In both situations, the DNA-RNA hybrid activates DNA synthesis by displacing the non-transcribed DNA strand, thus exposing potential recognition sites of a helicase/primase that can simultaneously drive the replication fork forward and synthesize primers for the lagging strand (Marians, 1992). Importantly, the formation of the persistent hybrid between the RNA and the template DNA is necessary for ColEI replication. In particular, the interaction between the dC stretch in the DNA template strand and the rG stretch in the RNA is essential for the formation of the stable R-loop (Masukata and Tomizawa, 1990). Interestingly, the efficiency of persistent hybrid formation depends on the rate of elongation of the transcript, suggesting that its success requires the formation of a particular DNA-RNA structure at a particular time during transcription (Masukata and Tomizawa, 1990).

Another long-known example of R-loop-mediated DNA synthesis occurs early during bacteriophage T4 infection (Mosig, 1987). Several putative origins of replication have been identified in T4 and the best characterized (*oriF* and *oriG*) consist of two components: a middle-mode promoter and a downstream DNA unwinding element (DUE; Carles-Kinch and Kreuzer, 1997). In a first step, transcription initiates from the promoter; in a second step, a persistent DNA-RNA hybrid is formed within the DUE region, providing the primer for leading-strand synthesis at the 3′end generated either by RNA polymerase or by RNAse cleavage. Alternatively, the R-loop structure allows T4 primase to synthesize RNA primers on the single-stranded non-template strand (Belanger and Kreuzer, 1998). It is possible that either mechanism can be used to prime leading-strand DNA synthesis depending on protein availability as reported for plasmid ColEI replication (Dasgupta et al., 1987; Masukata et al., 1987). The finding that non-origin plasmids are efficiently replicated *in vitro* by the T4 replisome, providing they carry a preformed R-loop within the DUE region, strongly implies that the R-loop itself supplies the signal for replisome assembly (Kreuzer and Brister, 2010).

Mitochondrial DNA replication at the leading-strand origin is also coupled to transcription through the formation of an R-loop (Chang and Clayton, 1985; Chang et al., 1985). The critical features of this origin are conserved from *Saccharomyces cerevisiae* to humans and include a promoter and a downstream short GC-rich cluster. *In vitro* transcription studies demonstrated that a short rG-dC sequence is the only necessary and sufficient *cis* element required for stable hybrid formation, although its efficiency depends on transcription by mtRNA polymerase and close proximity of the site of transcription initiation to the GC-rich cluster (Xu and Clayton, 1995). Once made, the RNA of the R-loop can serve as an effective primer for elongation by POLG, the mtDNA polymerase. These findings are reminiscent of those described for ColEI replication (Masukata et al., 1987), indicating that stable R-loop formation depends on C-rich clusters on template DNA. Additionally, the highly conserved nature of this essential template sequence element suggests that its role in stabilizing R-loop formation is ancient and likely pervasive in mitochondrial genomes (Xu and Clayton, 1995). More recently, an unorthodox mechanism of mtDNA replication involving long stretches of preformed RNA hybridized to the template-lagging strand has been described (Reyes et al., 2013). These long tracts of RNA are not products of on-going transcription and are therefore not directly related to the R-loops discussed here.

Replication of the *E coli* chromosome can also initiate by a mechanism involving R-loops in RNAse HI knock-out cells (Asai and Kogoma, 1994), and likely in wild-type cells under certain specific conditions such as entry into stationary phase or replication after DNA damage (Hong et al., 1996; Camps and Loeb, 2005; Wimberly et al., 2013). During this alternative mode of replication, named constitutive stable DNA replication (cSDR), RNAse HI-deficient cells initiate oriC-independent replication from multiple chromosomal sites termed *oriKs*. This results in global alterations of replication fork migration patterns, frequently in the opposite direction to normally initiated *oriC* synthesis and converging replication forks meeting in unusual places around the chromosome (Maduike et al., 2014). Notably, *E. coli* cells with a reduced capacity to remove R-loops display SOS constitutive phenotypes and an increase in hotspots for homologous recombination at the chromosomal terminus region flanked by the *Ter* sites (Nishitani et al., 1993; Horiuchi et al., 1994; Hong et al., 1995). It should be noted, however, that R-loop formation during cSDR occurs by transcript invasion, in contrast to the co-transcriptional R-loops generated in ColE1 replication (Kogoma, 1997).

## Replication Origin Specification in Eukaryotes

In eukaryotic genomes, DNA synthesis initiates from multiple replication origins. Accurate duplication of the genetic material depends on a reliable mechanism that ensures that any given origin fires at most once per cell cycle by restricting “licensing” to late mitosis and “activation” to S phase. At the anaphase to telophase transition the origin recognition complex (ORC) is recruited to replication origins; then, licensing rapidly occurs through the loading of the double hexameric minichromosome maintenance (MCM) complex together with other proteins such as Cdc6 and Cdt1 to form the pre-replication complex (pre-RC). Activation occurs when the pre-RC is converted to the pre-initiation complex (pre-IC) by the assembly of several replication factors facilitating the switch of the MCM complex to the active CMG (Cdc45-MCM-GINS) helicase during S-phase. The formation of the pre-IC depends on two protein kinases, cyclin-dependent kinase (CDK) and Dbf4-dependent kinase (DDK) that would ultimately trigger the unwinding of the origin DNA and the establishment of bidirectional replication forks (Siddiqui et al., 2013; Tanaka and Araki, 2013; and references therein). Replication initiation has been recently reconstituted *in vitro* with purified replication proteins, thus defining the initiation factors required for regulated eukaryotic DNA replication (Yeeles et al., 2015). Since there are far many more licensed ORIs than activated in each S phase, this is interpreted as a fail-safe mechanism used by cells to cope with replication stress (Ge et al., 2007; Ibarra et al., 2008). While the role of ORC in the pre-RC assembly at replication origins is conserved among various eukaryotes, the mechanism of origin recognition by ORC seems different across eukaryotic species (Bell et al., 2013). For example, ORC can be targeted to replication initiation sites by sequence-specific interaction as for *S. cerevisiae* (Marahrens and Stillman, 1992), or by non-specific binding to AT-rich sequences as for *Schizosaccharomyces pombe* or Drosophila (Kong and DePamphilis, 2001; Vashee et al., 2003). In addition, interactions can occur through sequence-specific binding proteins as for Drosophila and at certain loci in rat and human cells (Beall et al., 2002; Minami et al., 2006; Thomae et al., 2008), or through RNA-binding as in the case of 26T RNA during rDNA amplification in *Tetrahymena thermophila* (Mohammad et al., 2007) or during Epstein-Barr virus replication (Norseen et al., 2008).

Although no DNA replication activity similar to *E. coli* cSDR has been found in eukaryotes, transcription activity is strongly associated with initiation of DNA replication in mammalian systems. Specifically, genome-wide maps of replication origins in mouse and human cells showed that the most efficiently activated and more conserved origins across all cell types examined are those associated with CpG island promoters (Sequeira-Mendes et al., 2009; Cayrou et al., 2011; Besnard et al., 2012; Picard et al., 2014). Intriguingly, these genomic analyses revealed that a G-rich repeat element with the potential to form G-quadruplex structures (G4) was present in most of the replication origins in mouse and human cells (Besnard et al., 2012; Cayrou et al., 2012). The role of G4 structures in origin specification is not clear, however, recent evidence demonstrated that some G4 motifs could stimulate replication initiation (Valton et al., 2014). An interesting possibility is that G4 structures could mediate ORC recruitment to initiation sites. This notion is supported by *in vitro* binding assays demonstrating that the human ORC has affinity for G4 motifs through a specific domain in the ORC1 protein. Notably, ORC1 affinity for G4 motifs is almost negligible on double-stranded DNA but it is highly increased when G4 motifs are present on RNA or single-stranded DNA (Hoshina et al., 2013). As many CpG island promoters are prone to R-loop formation upon transcription (Ginno et al., 2012, 2013), this could provide a possible mechanism by which single-stranded G4 structures are formed within the origin region early during the cell cycle generating a potential substrate for ORC1 binding by the end of mitosis. To test this possibility, we analyzed the co-occurrence of ORC1 binding sites and R-loops at CpG island-origin regions in human cells.

## Association of ORC1 Binding Sites and R-loops in Human Cells

As mentioned above, CpG island origins are the most conserved and highly-efficient origins in the mammalian genome, as determined by the increased levels of associated short nascent strands (SNS) detected either by array hybridization signals (Sequeira-Mendes et al., 2009; Cayrou et al., 2011) or by sequencing read depth relative to the length of the origin (Besnard et al., 2012; Picard et al., 2014). Consistent with their higher firing activity, 37% of the ORC1 binding sites identified genome-wide in HeLa cells were associated with CpG island promoters, and their corresponding ORC1-ChIP signal was significantly stronger (Dellino et al., 2013). Nevertheless, the large majority of the non-CpG island origins identified by SNS-Seq do not co-localize with ORC sites (Dellino et al., 2013; Picard et al., 2014). We therefore selected for our study the subset of CpG island promoters enriched in SNS (Besnard et al., 2012) that were positive for ORC1 binding (Dellino et al., 2013) in datasets derived from the same cell line. This set of CpG island-origins (1,661 from a total of 9,864 TSS-associated CpG island promoters identified at the UCSC database-hg19), although very restrictive, confidently comprises *bona-fide* highly efficient constitutive replication origins. Notably, this core of CpG island-origins display the highest density of G4 motifs (Picard et al., 2014), supporting the notion that G4 motifs can play a role in the control of origin selection in the human genome.

**FIGURE 1:**
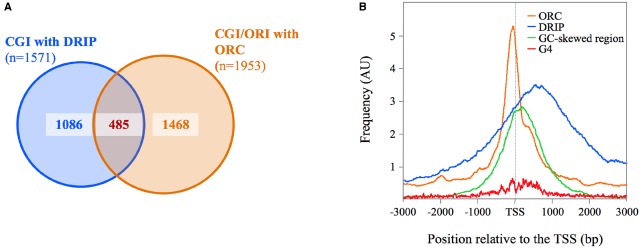
**Association between R-loops and ORC1-binding sites at CpG island-origins in human cells. (A)** Venn diagrams illustrating the association between R-loops (defined as consensus DRIP peaks in Ginno et al., 2013) and ORC1-binding sites at origin regions (defined by intersecting ORC1 binding sites from Dellino et al., 2013 with SNS-Seq data from Besnard et al., 2012) at CpG island promoters in human cells (UCSC database hg19). See text for details. Alignment of the DRIP-Seq or SNS-Seq reads to the hg19 build was carried out using BWA (Li and Durbin, 2009), and peak calling was done using MACSv2 (Zhang et al., 2008). For DRIP-seq, peaks were called using all mapped reads, enforcing a greater than fivefold enrichment above input as described (Ginno et al., 2012). **(B)** Distribution of ORC1-binding sites (orange lines), DRIP peaks (blue lines) and G4 motifs (red lines) at the CpG-island origin set defined in **(A)**. Composite profiles were generated by plotting hits per base over 6 kb for 485 CpG island regions centered at their TSS (defined as the 5′-end of RefSeq genes) normalized by the total hits over the whole genome. G4 positions were determined applying Quadparser on hg19 and specifying a loop size of 1–7 nucleotides between 4 tracks of GGG or CCC (Huppert and Balasubramanian, 2005). The green line represents the localization of the GC-skewed region of the analyzed CpG islands from Ginno et al. (2012).

CpG island promoters, regardless of their enrichment in CpG dinucleotides, remain unmethylated in normal tissues (Illingworth and Bird, 2009). The majority of these unmethylated CpG island promoters show significant strand asymmetry in the distribution of guanines and cytosines, a property known as GC skew (Ginno et al., 2012, 2013). The Chédin lab found that nearly 75% of CpG island promoters (35% of al human promoters) displayed a positive GC skew, signifying that the non-template strand for transcription has an excess of G over C residues (Ginno et al., 2012). To test whether GC skew confers the ability to form R-loops upon transcription, the authors performed a genome-wide identification of R-loop by DRIP-Seq (DNA-RNA immunoprecipitation with the R-loop-specific S9.6 antibody coupled to deep-sequencing). A total of 4,181 DRIP-Seq peaks were identified in two complementary assays of DNA fragmentation, from which nearly 40% (*n* = 1571) mapped to GC-skewed CpG island promoters (Ginno et al., 2013). We chose this stringent DRIP peak dataset to study the association between R-loops and ORC1 binding sites. We found that 30% (*n* = 485) of the CpG islands displaying R-loops showed ORC1 binding just upstream of their TSS, compared with the 6% expected by chance (Figure 1A). ORC1 and DRIP signals overlap with each other precisely at the 1 kb GC-skewed footprint defined for this set of CpG islands (Ginno et al., 2012; Figure 1B). It should be noted that the exact localization of the R-loops could not be extrapolated from the DRIP profiles as the resolution of the DRIP-Seq depends on the distribution of the restriction sites used to fragment the genome at each of the CpG islands analyzed.

These overlaps likely represent a great underestimate giving the fact that DRIP profiling experiments and origin mapping experiments were performed in different cell types (Ntera2 cells *versus* Hela cells), as well as the extremely stringent criteria used in selecting the significant regions from the various datasets for the analysis. Indeed, visual inspection of the sequencing reads on the UCSC genomic browser showed that many of the CpG island-origins defined by SNS enrichment displayed an ORC1 signal just below the threshold of significance. Similarly, several of the ORC1 positive CpG islands co-localize with R-loops defined in one set of DRIP data, but not in the other, due to the non-overlapping distribution of the cleavage sites of the restriction enzymes used to fractionate the genome in the two experiments analyzed. Nevertheless, these analyses show that R-loops co-localize with ORC1 binding sites within the same CpG island region at a fraction of the most efficient replication origins in human cells. More importantly, G4-motifs enrichment parallels the ORC1 signal at this CpG island-origin set (Figure 1B), consistent with the idea that G4 structures can mediate ORC recruitment to these specific sites.

By which mechanism could R-loops mediate ORC recruitment? The simplest possibility is that R-loop formation facilitates the generation of G4 structures on the displaced single-stranded non-template DNA strand that can target the ORC. Another possibility is that the ORC could be directly tethered to G4 structures formed on the RNA component of the hybrid. Indeed, EBNA1-ORC binding during EBV replication occurs through G-rich RNA and this binding is disrupted by G4-interacting drugs (Norseen et al., 2009). Finally, it is also possible that the ORC can bind hybrid G4 structures formed between the G-rich RNA and the G-rich displaced DNA strand generated by the R-loop. These hybrid G4 structures occur at the DNA replication leading-strand origin (OriH) in mammalian mitochondria and seem to regulate transcription termination at the replication-priming site (Wanrooij et al., 2012; Zheng et al., 2014). Any of these possible scenarios fulfill the affinity requirements described for ORC1 in *in vitro* assays (Hoshina et al., 2013) and would imply that G4 structures should persist through mitosis to mediate ORC recruitment. G4 structures formed on the displaced DNA strand could, in turn, stabilize the R-loops (Aguilera and García-Muse, 2012) and possibly inhibit nucleosome assembly at those sites (Wong and Huppert, 2009). Interestingly, we recently reported that ORC1 binding sites at efficient CpG island origins in human cells occupy the position marked by unstable nucleosome particles composed by H3.3/H2A.Z double-histone variants (Lombraña et al., 2013). Altogether, this suggests that stable R-loop formation at CpG island promoters could contribute to the generation of a permissive environment for ORC recruitment by, on one hand, exposing single-stranded DNA or RNA G4 structures and, on the other hand, by facilitating the assembly of labile nucleosome particles at those sites, thus preventing these regions from being occupied by adjacent stable nucleosomes or non-specific factors. It is worth mentioning that the group of CpG island promoter-origins analyzed here mainly comprises strong promoters driving high transcriptional outputs mapping on gene-poor chromosomes (Ginno et al., 2013). As gene-poor genomic regions tend to be depleted from replication origins (Besnard et al., 2012; Picard et al., 2014), it is tempting to speculate that R-loop-mediated ORC recruitment, or G1-formed R-loops, could be one of the multiple factors influencing the choice of origins to be fired to initiate DNA synthesis during S-phase (Renard-Guillet et al., 2014). This mechanism could be especially relevant at this subset of CpG islands as a means to increase the probability of firing within genomic environments otherwise scarce in replication initiation sites. Indeed, replication origin paucity has been proposed as a major cause of the increased instability observed in common fragile sites in human cells (Letessier et al., 2011; Ozeri-Galai et al., 2011). Another interesting consideration is that CpG island firing activity during S phase generates short re-replicated DNA fragments (Gómez and Antequera, 2008) precisely derived from the position occupied by labile nucleosome particles where the ORC binds (Lombraña et al., 2013). Whether this phenomenon is related to R-loop formation or whether R-loop dysregulation can lead to extensive overreplication and genomic instability awaits elucidation.

The above scenario is consistent with the view that DNA replication origins in mammals are not unique entities with defined properties (Antequera, 2004; Gilbert, 2010; Méchali, 2010; Sequeira-Mendes and Gómez, 2012). On the contrary, increasing evidence suggests that the origin structure consists of redundant binding sites made accessible to the ORC by local chromatin conformation associated with gene transcription, including R-loop formation. This opportunistic coupling of DNA replication initiation to transcription presents the advantage that it links DNA synthesis to cellular physiology, enhancing the robustness of the replication program and, at the same time, allowing faster adaptation to environmental or developmental changes (Sequeira-Mendes and Gómez, 2012).

## Conclusion

The proposal that R-loop formation at CpG islands can contribute to replication origin specification in human cells by exposing single-stranded DNA or RNA G-quadruplex structures strengthens the view that sequence-driven DNA structures may represent a new layer of regulatory information. Given the increasing evidence that abnormal R-loop accumulation can compromise genome stability, understanding how cells prevent the negative effects of R-loops yet allowing their positive effects is a challenge for the years to come. In the particular case discussed here, failure in the strict control of replication origin activation at CpG islands *via* R-loop dysregulation can lead to aberrant DNA replication, one of the hallmarks of cancer cells.

### Conflict of Interest Statement

The authors declare that the research was conducted in the absence of any commercial or financial relationships that could be construed as a potential conflict of interest.
